# Education in Medicine: Moving the Boundaries to Foster Interdisciplinarity

**DOI:** 10.3389/fmed.2016.00015

**Published:** 2016-04-08

**Authors:** Michel Goldman

**Affiliations:** ^1^Institute for Interdisciplinary Innovation in healthcare, Université libre de Bruxelles, Brussels, Belgium

**Keywords:** translational medicine, precision medicine, education, regulatory science, patient centricity, trial designs

## Introduction

Healthcare is at the verge of a revolution. We are entering the era of “precision medicine” in which prevention and treatment of diseases will be tailored according to the characteristics of each patient and the in-depth characterization of the pathologic processes underlying human disorders (1). This novel approach of medical care will include, among others, powerful computational analyses of large sets of biological and clinical data collected *via* electronic health records and mobile health technologies. Indeed, risk to develop specific disorders, and chance to respond to particular preventive or therapeutic measures, will be determined by individual profiles emerging from the integrated collection of clinical, genetic, environmental, and lifestyle data. This new knowledge will result in novel diagnostic and therapeutic tools, such as genomics-based tests, interventional imaging techniques, innovative medical devices, and cell-based or gene-based therapies, in addition to more efficient and safer (bio)pharmaceuticals.

In this context, medicine will converge more and more with other life sciences, physical sciences and engineering sciences, which would require new models of collaborative innovation. Furthermore, innovation in healthcare will necessitate more than scientific and technological advances. In order to fill the gap between lab bench and patient bedside, new approaches for product development, regulation, market access (pricing/reimbursement), and patient access and adoption will become increasingly important.

Translational medicine can be defined as the interdisciplinary science that will cover this continuum, from basic research to preclinical and clinical research, development of new medicines and medical devices, and ultimately patient-centric care. Interestingly, this is not a unidirectional path as very often it might be necessary to go back to research when unexpected findings are made after introduction of a new product on the market. The holistic approach inherent to translational medicine is essential to address the major public health challenges that our societies are facing, such as Alzheimer’s dementia, the diabetes’ epidemics, antimicrobial resistance, or the development of novel cancer therapies.

Irrespective of their specific role in the healthcare system of the future, physicians and other healthcare providers, scientists, managers, regulators, and policy makers will need knowledge in translational medicine to be able to contribute to interdisciplinary endeavors. They will also have to develop the skills necessary to address organizational, business, and management issues in this complex sector.

Academia represents an ideal environment to develop the educational and training programs needed to prepare the next generation of healthcare professionals to this challenge. Indeed, universities and other academic institutions offer advanced expertise in the multiple domains to be considered for the setup of cross-disciplinary research and education. Academic programs should be developed in close collaboration with the industrial sector, which is already investing in continuous professional development and lifelong learning activities relevant to translational medicine.

Herein, we underline key elements of precision medicine that need to be considered in the development of interdisciplinary educational programs.

## Development of a New Taxonomy of Diseases

In medical practice, the most commonly used taxonomy system is the International Classification of Diseases established more than 100 years ago by the World Health Organization. Although this classification resulted in significant medical advances and the development of several blockbuster drugs, it is not fit-for-purpose for precision medicine. In order to tailor therapies according to molecular changes underlying pathological processes, it is essential to integrate clinical data with information derived from genomics and other “omics” sciences, i.e., proteomics, metabolomics, and transcriptomics. Imaging data should also be included in the new classification of diseases. The cancer field is currently the one in which the benefit of such “systems medicine” approach to optimize the treatment of patients is best established. The development of this new taxonomy of diseases will depend on the exploitation of “Big Data” that will have to be appropriately collected through different tools including electronic health records. In this respect, the development and use of standards will be the key to ensure data interoperability (2). Likewise, it will be essential to ensure data accuracy and prevent publication of unreliable information.

## Developing New Trials Designs

There is a clear need to develop new types of clinical trials to address the limitations of classical randomized controlled trials that are characterized by rigid protocols. Adaptive trial designs elicit particular interest from investigators, industry, and patients because they offer opportunities to prospectively plan protocol’s modifications on the basis of accumulated data, without jeopardizing the validity of the study’s conclusions. Possible adaptations may involve drug dosage, allocation of treatments, addition or deletion of treatment arms, combination of therapies, adjustment of statistical hypotheses, etc. Indeed, sophisticated statistical analyses and powerful computing platforms are necessary to ensure the reliability of these adaptive trial designs which help to select most promising therapies early, reduce the number of patients enrolled, and overall speed up the process of development. Precision medicine will greatly benefit from adaptive designs, especially those that use biomarkers for enrichment in a specific patient population for early detection of disease.

## Moving Regulatory Science Forward

Regulators are expected to make recommendations on therapies according to the balance between the benefits they provide and the potential adverse events they might induce. Until recently, regulators were merely considered as gatekeepers of patient safety. Nowadays, they must become active partners in the efficient implementation of precision medicine. This will require adaptations of regulatory policies and guidelines according to the so-called “regulatory science,” which includes novel tools to assess drug safety preclinically and new approaches to assess drug effectiveness under real-life conditions. Furthermore, regulatory bodies will need to adapt their licensing schemes in order to build new pathways to bring innovative therapies to patients more efficiently (3). This is the focus of a remarkable multi-stakeholder international collaboration, the Medicines Adaptive Pathways to Patients (MAPPS) initiative (http://bit.ly/1W7GLAt).

## Ensuring Patient Access

One of the greatest challenges to introduce precision medicine in the standard of care is to make it affordable to individuals and society. The ongoing debate about the prices of the new curative therapies for hepatitis C and the immune check point inhibitors, which dramatically improve the treatment of melanoma and other cancers, illustrate the need for new approaches to assess the balance between cost and value in healthcare. The development of targeted therapies is complex not only scientifically but also from an economic standpoint. With the advent of tailored care, numbers of patients eligible for breakthrough therapies and corresponding market size are markedly reduced. As the era of blockbusters is over, industry has to adopt new schemes to get return on their investments in research and development and to make pharmaceutical innovation profitable. In parallel, payers must ensure the sustainability of healthcare costs. Methodologies that facilitate adjustment of drug pricing on the basis of objective criteria will need to be developed, especially those based on patient outcomes. Clearly, health economics and management must be included in the interdisciplinary programs designed to move precision medicine forward (4).

## Patient Centricity

The assessment of health outcomes will be essential to capture the value of precision medicine and make it sustainable. Patients have a key role to play in this assessment, as recognized by industry and regulators. What patients expect from new therapies must be considered in the design of clinical studies. Of particular importance is their evaluation of the balance between risks and benefits, even if this dimension needs to integrate the impact of a self-centered perspective – inherent to the patient’s position. Research, education, and training in social and behavioral sciences will be important to ensure appropriate communication and dialog with patients about the key questions related to health outcomes and effectiveness of new therapies.

## Concluding Remarks

Collaboration between healthcare stakeholders will be essential to address the complex scientific, regulatory, societal, and economic challenges related to precision medicine. This will require reconciling and aligning the interests of private for-profit companies and publicly funded not-profit institutions around patients’ needs and expectations. Recent years have witnessed the establishment of a number of public private partnerships and research consortia with this objective, including the Innovative Medicines Initiative (IMI) in Europe, the Critical Path Initiative, the Foundation for the NIH and the Accelerating Medicines Partnership in the United States, and CQDM in Canada. They represent the natural instruments to assemble the critical mass of expertise and resources necessary to implement the joint endeavors that should result in new therapies for major unmet needs, including brain disorders, antimicrobial resistance, diabetes, immune-inflammatory diseases, and cancer. Although meaningful results have already been achieved, future advances will depend on innovative educational programs that will provide the next generation of healthcare professionals with sound and robust bases for interdisciplinary patient-focused approaches and will develop their mindset for collaboration (4). Scientific journals have an important role in this respect. As an open access journal with digital platforms that aim at catalyzing collaboration, Frontiers in Medicine is fully committed to contribute to this endeavor.

## Author Contributions

MG conceived and wrote the paper.

## Conflict of Interest Statement

The author declares that the research was conducted in the absence of any commercial or financial relationships that could be construed as a potential conflict of interest.
